# Prediction of pathological complete response to neoadjuvant chemotherapy in breast cancer using a deep learning (DL) method

**DOI:** 10.1111/1759-7714.13309

**Published:** 2020-01-16

**Authors:** Yu‐Hong Qu, Hai‐Tao Zhu, Kun Cao, Xiao‐Ting Li, Meng Ye, Ying‐Shi Sun

**Affiliations:** ^1^ Key laboratory of Carcinogenesis and Translational Research (Ministry of Education/Beijing), Department of Radiology, Peking University Cancer Hospital and Institute Beijing China; ^2^ Radiology Peking University Cancer Hospital & Institute Beijing China; ^3^ Radiology Beijing China

**Keywords:** Breast cancer, DCE‐MRI, deep learning, pathologic complete response

## Abstract

**Background:**

The aim of the study was to develop a deep learning (DL) algorithm to evaluate the pathological complete response (pCR) to neoadjuvant chemotherapy in breast cancer.

**Methods:**

A total of 302 breast cancer patients in this retrospective study were randomly divided into a training set (*n* = 244) and a validation set (*n* = 58). Tumor regions were manually delineated on each slice by two expert radiologists on enhanced T1‐weighted images. Pathological results were used as ground truth. Deep learning network contained five repetitions of convolution and max‐pooling layers and ended with three dense layers. The pre‐NAC model and post‐NAC model inputted six phases of pre‐NAC and post‐NAC images, respectively. The combined model used 12 channels from six phases of pre‐NAC and six phases of post‐NAC images. All models above included three indexes of molecular type as one additional input channel.

**Results:**

The training set contained 137 non‐pCR and 107 pCR participants. The validation set contained 33 non‐pCR and 25 pCR participants. The area under the receiver operating characteristic (ROC) curve (AUC) of three models was 0.553 for pre‐NAC, 0.968 for post‐NAC and 0.970 for the combined data, respectively. A significant difference was found in AUC between using pre‐NAC data alone and combined data (*P* < 0.001). The positive predictive value of the combined model was greater than that of the post‐NAC model (100% vs. 82.8%, *P* = 0.033).

**Conclusion:**

This study established a deep learning model to predict PCR status after neoadjuvant therapy by combining pre‐NAC and post‐NAC MRI data. The model performed better than using pre‐NAC data only, and also performed better than using post‐NAC data only.

**Key points:**

Significant findings of the study.

It achieved an AUC of 0.968 for pCR prediction. It showed a significantly greater AUC than using pre‐NAC data only.

What this study adds

This study established a deep learning model to predict PCR status after neoadjuvant therapy by combining pre‐NAC and post‐NAC MRI data.

## Introduction

Neoadjuvant chemotherapy (NAC) has been established as the first‐line treatment of locally advanced breast cancer (LABC), in order to reduce tumor size, downstage the disease, control the potential metastases, and potentially increase the chance of breast‐conserving surgery.^1^, ^2^, ^3^ Pathologic complete response (pCR), defined as complete resection of breast tissue without invasive tumor cell remnants, is the ideal result of NAC which could predict a good prognosis.^4^, ^5^, ^6^ Accurate determination of the achievement of pCR is of great significance to improve patients' treatment enthusiasm, increase breast‐conserving confidence, reduce anxiety and improve quality of life.

Multiple studies have shown that dynamic contrast‐enhanced magnetic resonance imaging (DCE‐MRI) has higher predictive power in predicting residual tumor size after NAC, compared with the conventional methods including clinical breast examination, mammography, and ultrasound.^7^, ^8^ Recent developments in radiomics have shown potentials in the prediction of pathological results by radiological data.^9^, ^10^ Deep learning makes it possible to automatically extract features from an image without the necessity of feature predefinition.^11^, ^12^

In previous studies, only pre‐NAC MRI data were used and the area under receiver operating characteristic (ROC) curve (AUC) ranged from 0.78 to 0.86.^9^, ^10^, ^12^ Actually, the structural and functional change in tumor microenvironment after NAC can reflect the therapeutic response effect of patients.^13^, ^14^ Therefore, we suspected that more accurate prediction could be achieved after NAC when post‐NAC MRI data were also available, but no relevant literature has been found.

In this study, we combined pre‐NAC and post‐NAC DCE‐MRI images of LABC into a convolutional neural network for better prediction of pCR status after NAC. The network was designed with inclusion of molecular types into fully connected layer. The DL model was trained on 244 participants and tested on 58 participants.

## Methods

### Patients

An institutional review board‐approved retrospective review of our database from January 2015 to December 2016 identified 316 locally advanced breast cancer patients who had (i) undergone breast MRI prior to the initiation of NAC and surgery, called baseline (pre‐NAC) and after treatment (post‐NAC), (ii) successfully completed adriamycin/taxane‐based NAC, and (iii) undergone surgical resection with available final postoperative pathology data. Patients were excluded if they had one of the following conditions: (i) history of other malignancy; (ii) insufficient MRI image quality; (iii) unilateral multifocal cancers; and (iv) incomplete immunohistochemical information. We enrolled 302 consecutive patients into the study. All participants were randomly divided into a training set (*n* = 244) and a validation set (*n* = 58). All patients received a schedule MR examination within seven days before NAC, called pre‐NAC and a post‐NAC examination within three days after NAC cycles.

### MRI scanning

MRI examinations were performed with a 1.5‐T MR scanner using a dedicated four‐channel phased array breast coil (Echospeed Plus with EXCITE II, GE Medical Systems, Milwaukee, USA). MRI protocols included a sagittal, 3D Vibrant SPGR sequence for dynamic imaging (TR = 6.4 ms, TE = 3.0 ms, TI = 7.0 ms, flip angle = 10°, slice thickness = 4 mm without any interslice gap, matrix size = 256 × 256, field of view = 20–22 cm, NEX = 1, ZIP2, and scan time per acquisition = 68 seconds); and an axial, fat‐suppressed, T1‐weighted pulse sequence with enhancement. The Vibrant sequence was continuously repeated six times, with one phase before and five phases after contrast enhancement for dynamic acquisition. The contrast agent (Gd‐DTPA) was injected into the antecubital vein by a power injector at a rate of 2.0 mL/second based on patient body mass (0.2 mmol/kg), followed by a saline flush. An initial fat‐saturated T1‐weighted precontrast scan was first collected. A first postcontrast scan was collected two minutes after contrast agent injection. Four subsequent postcontrast images were acquired at intervals of 90 seconds, resulting in five postcontrast images for each patient (t = 2, 3.5, 5, 6.5, and 8 minutes).

### Immunohistochemical analysis

Standard histopathological analysis was processed for the pathologic assessment of the response to NAC. Surgically resected specimens were fixed in 10% neutral buffered formalin and processed overnight in standard tissue processors. Slides were cut at 5 mm and stained using an automated staining system. The histopathological examination and analysis were performed by a pathologist (with eight‐years experience in breast pathology) who was blind to the radiological information. pCR was defined as an absence of invasive cancer in the breast surgical specimen and no cancer was found in the ipsilateral sentinel lymph node or resected nodes during axillary lymph node dissection (yPT0/isN0).^15^, ^16^

### Neoadjuvant treatment regimens

Human epidermal growth factor receptor 2‐negative (HER2−) patients received doxorubicin and cyclophosphamide every two weeks for four cycles followed by four cycles of paclitaxel. All HER2+ patients received treatment with docetaxel and trastuzumab every three weeks for six cycles. The HER2+ treatment regimen was also supplemented with pertuzumab and/or carboplatin on a patient‐by‐patient basis, depending on disease severity and availability at the time of treatment.

### Radiologist annotations

Pre‐ and post‐NAC MRIs were reviewed and analyzed by two experienced radiologists (one with 23‐years experience, one with five‐years experience) who were both blinded to the pathological results. The regions of interest (ROIs) were drawn manually with a 3D‐slicer (Version 4.8.1) (https://www.slicer.org) on each slice of tumor. On pre‐NAC MR image, ROIs were delineated along the contour of the tumor, containing the surrounding chords and burrs as visualized by the second phase of T1‐weighted images with Gadolinium enhancement. If a highly suspicious tumor signal was still visible on the second phase of enhancement (the early enhancement area), the post‐NAC delineation principle was the same as pre‐NAC. If no tumor signal was seen on the second phase of the enhanced scan (iso‐intensity signal compared with the normal breast parenchyma, no enhancement area), the ROIs were placed on the primary tumor region determined by the second phase of pre‐NAC enhancement.

### Inter‐ and intraobserver reproducibility evaluation

Inter‐ and intraobserver reproducibility of ROI delineation was evaluated by dice similarity coefficient (DSC). DSC was defined as: DSC = 2|X∩Y|/(|X| + |Y|), where X and Y were two different volumes. It equalled twice the number of elements common to both sets divided by the sum of the number of elements in each set. Interobserver reproducibility was evaluated on 30 randomly selected participants blindly delineated by Dr Cao and Dr Qu, respectively. To assess intraobserver reproducibility, Dr Qu repeated the ROI delineation on the 30 participants twice at two‐month intervals following the same procedure. A DSC greater than 0.85 was considered a good agreement.

### Deep learning and model construction

We resized all the lesion patches to a fixed size of 128 × 128 × 16 by zero‐padding. Augmentation was performed in the training set to increase the samples. Rotation by *i*/*n* × 360° (*i* = 1,2,…,*n*) was performed in the sagittal plane. *N* = 80 was used for pCR data and *n* = 60 was used for non‐pCR data, respectively to keep a balance between two classes. Signal normalization was performed in the six phases, scaling the maximum signal of enhancement to one and the minimum signal to 0. The smallest box that contained the whole lesion with 6 mm margin on each direction was created and inputted into the network.

**Figure 1:**
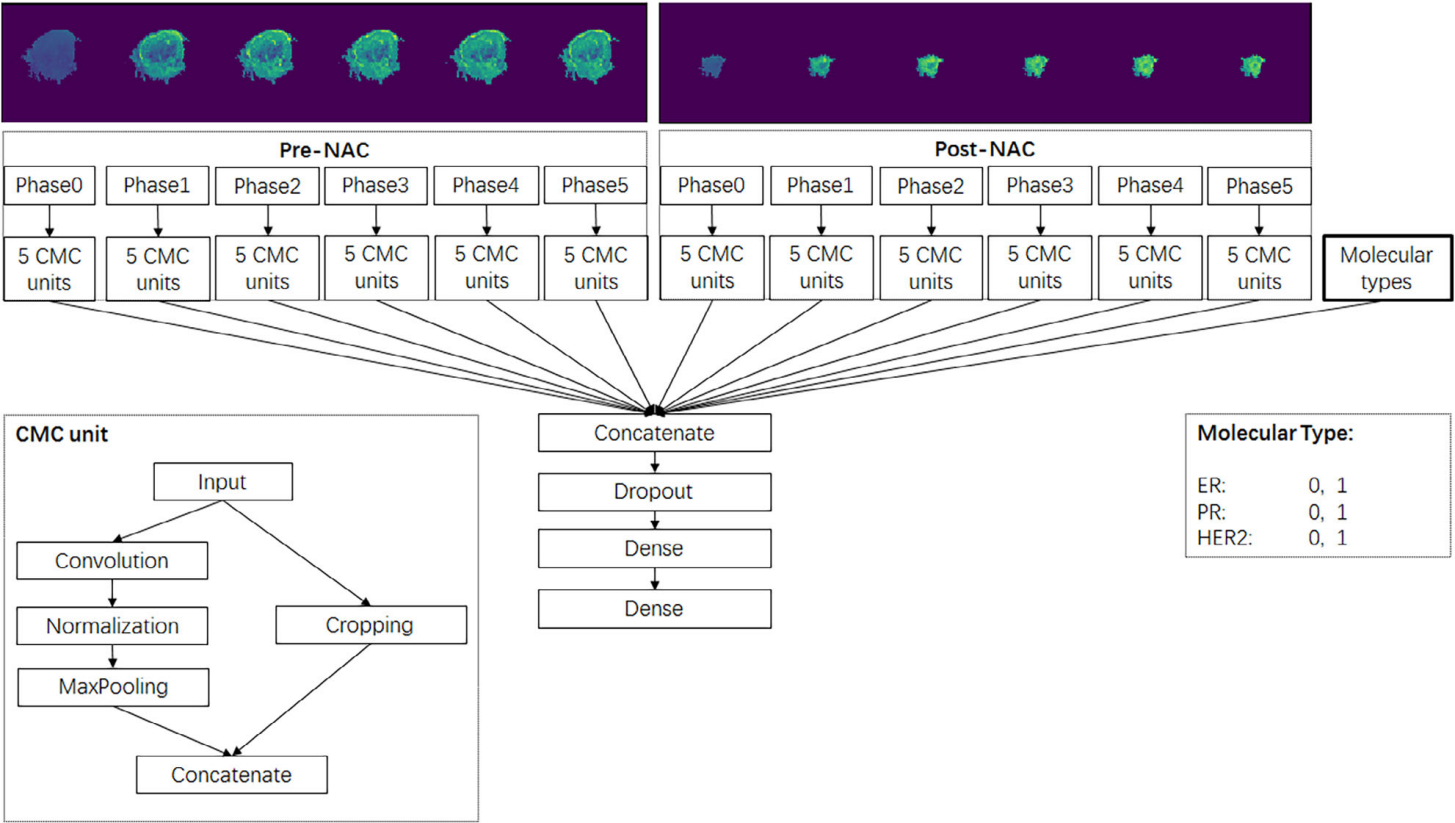
A multipath deep convolutional neural network architecture.

DL network was constructed using Python (V3.6) based on Keras (2.15) with TensorFlow (V1.4.0). It contained five repetitions of convolution and max‐pooling layers (CMC unit) and ends with three dense layers. In order to extract useful features and information from multiple phases of contrast enhancement before and after NAC treatment, we designed and trained a multipath convolutional neural network. The pre‐NAC model and post‐NAC model inputted six phases of enhancement from pre‐NAC and post‐NAC images, respectively. The combined model used 12 channels from six phases of pre‐NAC and six phases of post‐NAC images. All models above include three indexes of molecular type as one additional input channel. Figure 1 shows the structure of the network.

Each channel of image input was processed independently by a feature extraction unit and combined with molecular subtypes at the full‐connected layer. The feature extraction unit was composed of five repetitions of convolution/max‐pooling and cropping. While the max‐pooling layer downsampled the image, the cropping layer extracted the central part of the image. The concatenation of max‐pooling and cropping made it possible to extract features from different scales. The last layer was a Softmax layer which would output a class probability. Pathological results were used as ground truth.

The training set was split into four subgroups. Cross‐validation was performed by using three out of the four subgroups as training and the fourth one as validation to determine the hyperparameters, such as learning rate (selected from 1e−3, 3e−4, 1e−4, 3e−5, 1e−5),decay rate (selected from 0.1, 0.01, 0.001, 0.0001) and epochs (less than 10 000). After cross‐validation in the training set, the model with all four subgroups was trained with fixed hyperparameters. The model was finally tested on the validation set.

## Results

**Figure 2:**
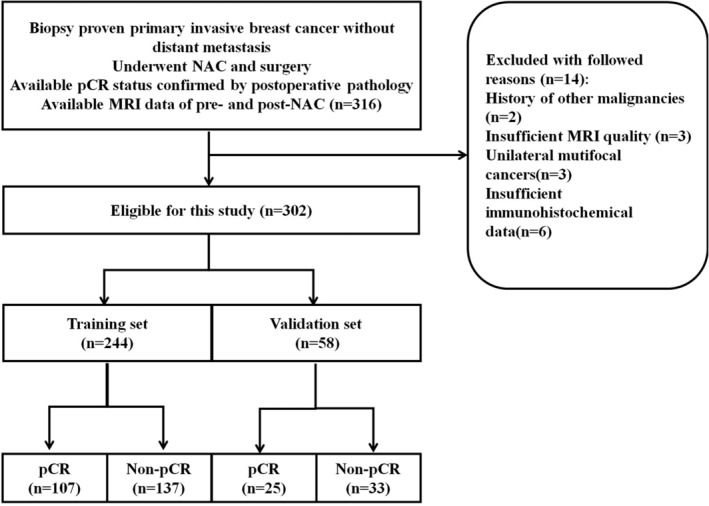
The inclusion and exclusion flowchart. There were 316 participants enrolled into this study and 14 were excluded.

As indicated in Figure. 2, 316 patients were enrolled consecutively and 14 patients were excluded according to the exclusion criteria. A total of 302 patients were finally included in this study and the characteristics of the patients are summarized in Table 1. No significant differences were found between the training set and validation set in terms of pCR prevalence (43.9% and 43.1% in the training and validation sets, respectively, *P* = 0.918). There were no significant differences between the pCR group and non‐pCR group in terms of age (*P* < 0.05). Additionally, some characteristics were significantly different between two groups, including pathological type, ER and PR status, both in the training and validation set. Meanwhile, pCR was found associated with HER2 status in the training set, but not in the validation set. The results suggested that the pCR prevalence were related to hormone receptor status and pathological type, which is consistent with previous studies.^17^, ^18^ The interobserver DSC was 0.92 for pre‐NAC ROI delineation and 0.88 for post‐NAC ROI delineation. The intraobserver DSC was 0.95 for pre‐NAC ROI delineation and 0.90 for post‐NAC ROI delineation.

**Table 1:** Characteristics of participants in the training and validation sets. A *t*‐test was used for continuous variables and chi‐square test for categorical variables

Characteristics	Training set			Validation set		
	pCR	Non‐pCR	*P*‐value	pCR	Non‐pCR	*P*‐value
	(*n* = 107)	(*n* = 137)		(*n* = 25)	(*n* = 33)	
Age (mean ± SD, years)	48.97 ± 10.43	49.34 ± 10.25	0.781	50.68 ± 8.87	47.82 ± 10.28	0.271
Pathological type (%)			0.015			0.026
Invasive ductal carcinoma， stage I	5	12		0	4	
Invasive ductal carcinoma， stage II	64	96		15	21	
Invasive ductal carcinoma， stage III	37	24		10	6	
Invasive papillary carcinoma	1	3		0	0	
Invasive lobular carcinoma	0	2		0	2	
ER (%)			0.000			0.002
Positive	54	106		8	24	
Negative	53	31		17	9	
PR (%)			0.000			0.014
Positive	63	110		11	25	
Negative	44	27		14	8	
HER2 (%)			0.000			0.604
Positive	68	36		10	11	
Negative	39	101		15	22	

**Figure 3:**
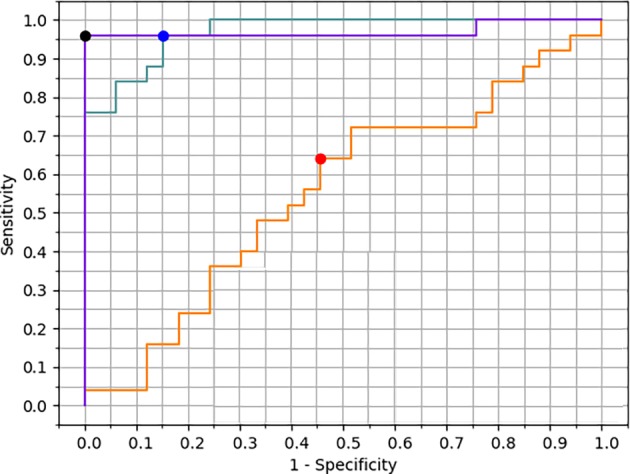
Receiver operating characteristic (ROC) curves of pre‐NAC, post‐NAC and combined model. The area under curve (AUC) was 0.553 (0.416–0.683) for the pre‐NAC model, 0.968 (0.885–0.997) for the post‐NAC model and 0.970 (0.887–0.997) for the combined model. The largest Youden index was used to set the cutoff value. (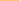) Pre‐NAC model (AUC = 0.553), (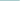) post‐NAC model (AUC = 0.968), (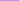) combined model (AUC = 0.970), (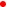) pre‐NAC model (balanced performance), (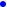) post‐NAC model (balanced performance), (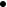) combined model (balanced performance).

After cross‐validation, we used the learning rate = 3e−5, decay rate = 0.01 and epochs = 2000 to train the whole training set. Probability of pCR was predicted on the validation set with a score between 0 and 1. Receiver operating characteristic (ROC) curve was plotted using the pathological result as the ground truth. Figure 3 shows the ROC curve of the three models. The area under the ROC curve (AUC) was 0.553 for pre‐NAC, 0.968 for post‐NAC and 0.970 for the combined model. A significant difference was found in AUC between using pre‐NAC data alone and using combined data (Z = 5.297, *P* < 0.0001). If the channel molecular type was excluded from the network, the accuracy of the combined model was slightly lower without significance (AUC = 0.942). Models containing molecular type channel were used in the following calculation.

The largest Youden index was used to set the cutoff value. The sensitivity (SEN), specificity (SPE), positive predictive value (PPV) and negative predictive value (NPV) are summarized in Table 2. Although the combined model had a similar AUC value with the post‐NAC model, the specificity of the combined model (SPE = 100%, 95% CI: 89.4–100.0) was larger than that of the post‐NAC model (SPE = 84.9%, 95% CI: 68.1–94.9). The positive predictive value of the combined model (PPV = 100%, 95% CI: 85.8–100.0) was greater than that of the post‐NAC model (PPV = 82.8%, 95% CI: 64.2–94.2) (χ2 = 4.569, *P* = 0.033).

**Table 2:** Performance of pCR prediction of 58 participants with locally advanced breast cancer in the validation set

	AUC	SEN %	SPE %	PPV %	NPV %
Pre‐NAC	0.553 (0.416–0.683)	72.0 (50.6–87.9)	48.5 (30.8–66.5)	51.4 (34.0–68.6)	69.6 (47.1–86.8)
Post‐NAC	0.968 (0.885–0.997)	96.0 (79.6–99.9)	84.9 (68.1–94.9)	82.8 (64.2–94.2)	96.6 (82.2–99.9)
Combined	0.970 (0.887–0.997)	96.0 (79.6–99.9)	100 (89.4–100.0)	100 (85.8–100.0)	97.1 (84.7–99.9)

NPV, negative predictive value; PPV, positive predictive value; SEN, sensitivity; SPE, specificity.

**Figure 4:**
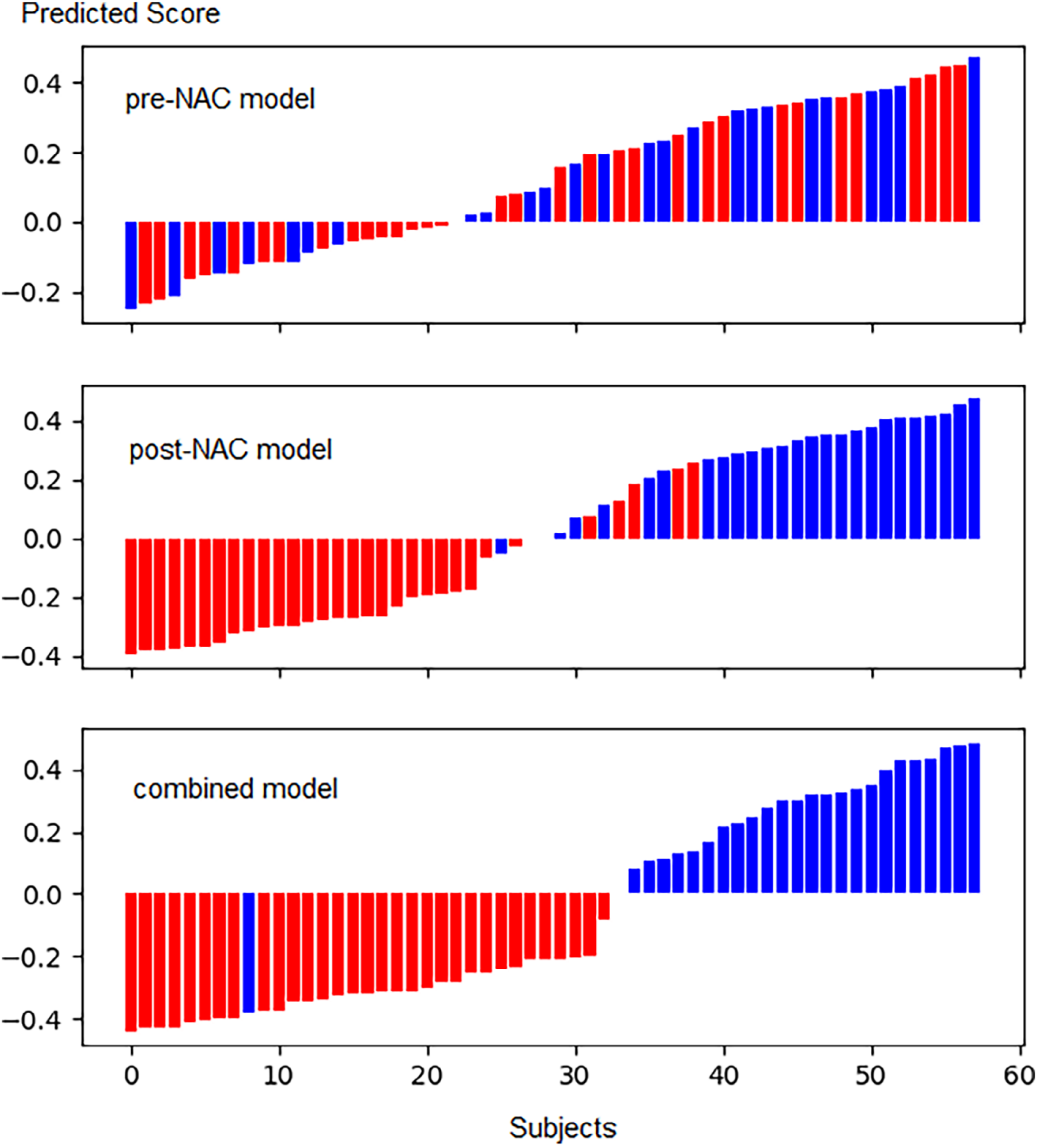
Predicted scores of 58 participants with locally advanced breast cancer in the validation set. Blue color indicates pCR proven by pathological analysis. Red color indicates non‐pCR proven by pathological analysis. Bars above 0 are pCR predicted by DL models. Bars below 0 are non‐pCR predicted by DL models.

Figure 4 shows the ordered predicted score minus the cutoff value. The red color indicates non‐pCR and blue color indicates pCR proven by pathological ground truth. The bars above the horizontal line indicates pCR and the bars below the horizontal line indicates non‐pCR predicted by the DL model.

**Figure 5:**
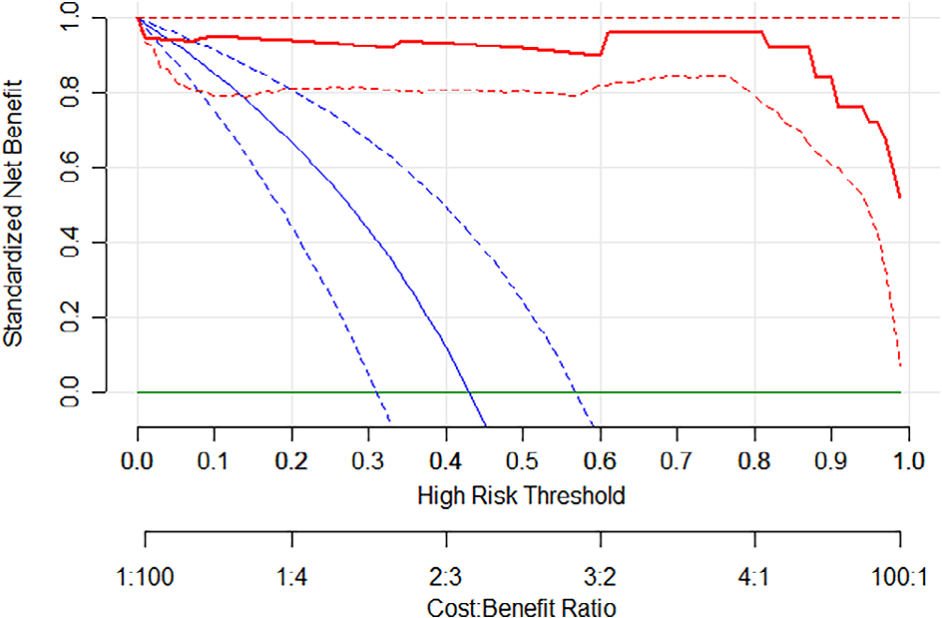
Decision curve analysis of deep learning model. The y‐axis measures the net benefit. The red line represents the deep learning model. The blue line represents the assumption that all patients achieved pCR after NCRT. The horizontal green line represents the assumption that no patients achieved pCR after NCRT. The net benefit was calculated by subtracting the proportion of all patients who were false positive from the proportion who were true positive, weighting by the relative harm of forgoing treatment compared with the negative consequences of an unnecessary treatment. Here, the relative harm was the ratio of the harm of false positive and the harm of false negative. It was calculated by Pt/(1 − Pt). Pt (threshold probability) is where the expected benefit of the treatment was equal to the expected benefit of avoiding treatment. A 95% confidence interval (dashed line) was determined by 1000 bootstraps. (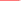) Combined DL model, (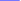) all and (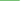) none.

Decision curve analysis (DCA) was performed to study the benefit of deep learning in Figure 5. The y‐axis measured the net benefit. The red line represents the deep learning model. The blue line represents the assumption that all patients achieved pCR after NCRT. The horizontal green line represents the assumption that no patients achieved pCR after NCRT. The net benefit was calculated by subtracting the proportion of all patients who were false positive from the proportion who were true positive, weighting by the relative harm of forgoing treatment compared with the negative consequences of an unnecessary treatment. Standardized net benefit scaled the net benefit into the range between 0 and 1. Here, the relative harm was the ratio of the harm of false positive and the harm of false negative. A 95% confidence interval (dashed line) was determined by 1000 bootstraps. The results shows that deep learning produced increased benefit in the whole range of risk threshold.

## Discussion

Accurate prediction of therapy response may change the treatment plan of LABC during NAC or before surgery. For example, if a bad response to a certain medicine could be predicted, the NAC plan of the patient may be changed as early as possible. If pCR could be predicted, more conservative treatment or surgery could be adopted. Several methods have been proposed by using radiomics or DL on pre‐NAC MRI data to predict pCR before the initiation of NAC. In several studies, the AUC ranged from 0.78 to 0.86.^9^, ^10^, ^12^ Unfortunately, to date, no radiomics or DL models have been used in clinical practice to change the clinical decision‐making of neoadjuvant therapy. This is probably due to the low accuracy that is still beyond an acceptable level in the clinic. In this study, we constructed a DL model by combining pre‐NAC and post‐NAC data and achieved an AUC of 0.98 and a PPV of 100%, which is much higher than previous studies for both traditional and functional MR imaging analysis.^19^, ^20^ It shows great potential in clinical application for pCR prediction after NAC. This accurate prediction may also have a positive impact on patients' morale for the following treatment and their confidence in breast conserving surgery.

Three models were compared in this study; pre‐NAC model, post‐NAC model and combined model. The combined model produced a significantly greater AUC than the pre‐NAC model (*P* < 0.0001). Although the AUC of the combined model was the same as that of the post‐NAC model, the 100% PPV of the combined model was significantly higher than that of the post‐NAC model (*P* = 0.033). PPV is an important index to evaluate the pCR prediction model. Results in this study suggested that the combination pre‐NAC and post‐NAC model was more accurate than either of them alone. The changes inside the tumor, including the changes in volume and enhancement features, are obviously reflected and should be taken into consideration.

The combined model misidentified one responder as non‐pCR. We found that case was ductal carcinoma in situ with residual tumor confirmed by postoperative pathology. The pre‐ and post‐NAC MRI images of the patient were again analyzed by the radiologist. In the post‐MRI images, no mass was found, but small patches of enhancement were still seen in the position of the original tumor bed, with the inflow pattern time signal curve, which may have been the cause of the model miscalculation.

Compared with radiomics, deep learning makes it possible to automatically extract features from an image without the necessity of feature predefinition. Studies solely based on pre‐NAC data show no significant difference between radiomics and DL for pCR prediction. As both pre‐NAC and post‐NAC data were included in this study, we suspected that DL might show advantages for its capability to extract a more sophisticated relationship between two sets of data. Although the inclusion of molecular type showed an insignificant increase in the AUC value for pCR prediction, it was reasonable to keep it as an input channel because molecular type has been proven as an indispensable index in clinical practice. To utilize molecular information, a deep convolutional neural network was designed to integrate molecular type inside the network structure. As the weights molecular type is optimized during training, this structure could be better than separating molecular information outside the network.

One limitation of our study was the use of a single imaging protocol for DCE MR imaging of pre‐ and post‐NAC. We considered that the T1WI and T1+C sequences of breast MRI were the most characteristic, and the multiparameter MRI deep learning model may require more training data. Another limitation of this study was that it was retrospective and single center. A prospective and multicenter study may help to construct a generalized prediction model appropriate for different clinical situations.

In conclusion, this study established a deep learning model to predict PCR status after neoadjuvant therapy by combining pre‐NAC and post‐NAC MRI data. The model showed a significantly larger AUC than using pre‐NAC data only, and also showed a significantly larger positive predictive value than using post‐NAC data only.

## Disclosure

No authors report any conflict of interest.
